# Risk Factors for the Progression or Regression to Diabetes or Normoglycaemia for Men with Impaired Fasting Glucose

**DOI:** 10.1155/jdr/9926306

**Published:** 2025-10-10

**Authors:** Jacob W. Harland, Natalie K. Hyde, Zoe Shih-Jung Liu, Courtney Swinton, Briana Spolding, Mark A. Kotowicz, Julie A. Pasco, Kara L. Holloway-Kew

**Affiliations:** ^1^Deakin University, IMPACT-Institute for Mental and Physical Health and Clinical Translation, Geelong, Australia; ^2^University Hospital Geelong, Barwon Health, Geelong, Australia; ^3^Department of Medicine, The University of Melbourne-Western Campus, Melbourne, Australia; ^4^Department of Epidemiology and Preventive Medicine, Monash University, Melbourne, Australia

**Keywords:** diabetes, impaired fasting glucose, men, progression, risk factors

## Abstract

**Aims:**

Reporting of impaired fasting glucose (IFG) prevalence and risk factors for progression to diabetes differs between studies, in part, due to the use of multiple definitions of the condition. We aimed to determine the prevalence of diabetes and IFG in men and identify risk factors for the progression to diabetes and regression to normoglycaemia.

**Materials and Methods:**

Participants were from the Geelong Osteoporosis Study. Diabetes was defined as fasting plasma glucose (FPG) ≥ 7.0 mmol/L, self-report of diabetes and/or use of antihyperglycaemic medication. IFG was defined using both American Diabetes Association (IFG-ADA: FPG 5.6–6.9 mmol/L) and World Health Organisation (IFG-WHO: FPG 6.1–6.9 mmol/L) criteria. Prevalence of hyperglycaemia at baseline (2001–2006, 1170 men, 20–97 years) was age-standardised to the 2006 Australian population. Multivariable logistic regression models (*n* = 416) were used to identify risk factors for the progression to diabetes and regression to normoglycaemia over 15 years.

**Results:**

Age-standardised prevalence of IFG-ADA was 18.2% (95% CI 15.7–20.7), 6.2% for IFG-WHO (95% CI 4.8–7.6) and 7.3% for diabetes (95% CI 5.8–8.8). Higher FPG, glycated haemoglobin, age, body fat percent and lower HDL cholesterol were associated with progression to diabetes, whereas younger age and higher HDL cholesterol were associated with regression. A 1.0 mmol/L increase in FPG resulted in a sixfold greater chance of progression to diabetes over follow-up (OR 6.44, 95% CI 2.97–13.94; *p* < 0.001). A prediction model containing age, FPG, HDL cholesterol and HbA1c predicted an optimal FPG cut point for progression of 5.3 mmol/L.

**Conclusion:**

This study reports cut points for predicting progression to diabetes that align with the ADA classification of IFG. These data may support future work investigating diabetes prevention strategies.

**Summary**

• Men with IFG under the WHO criterion have a greater chance of progressing to diabetes, although those under the ADA criterion still carry excess risk of progression.
• Prediction model containing FPG, age, HbA1c and HDL cholesterol produced an optimal FPG cut point of 5.3 mmol/L for predicting progression to diabetes in men.
• Risk factors for the progression to diabetes, higher fasting glucose and body fat percent are potentially modifiable and could be targets for prevention strategies.

## 1. Introduction

Diabetes mellitus is a chronic condition with substantial cardiovascular, neurological and renal comorbidities. In 2020–2021, 1.3 million Australians were estimated to have diabetes mellitus (Type 1 or 2), with most of these cases being Type 2 diabetes [1]. Between 2015 and 2016, an estimated AUD3.0 billion was spent on diabetes-related illness, which accounted for 2.3% of total disease expenditure by the Australian health system [2].

Impaired fasting glucose (IFG) is a state of hyperglycaemia that is higher than the normal values but below the threshold for diabetes using fasting plasma glucose (FPG). However, there is no universal diagnostic criterion; the American Diabetes Association (ADA) (5.6–6.9 mmol/L, 100–125 mg/dL) [3] and the World Health Organisation (WHO) (6.1–6.9 mmol/L, 110–125 mg/dL) provide differing cut points for FPG levels [4]. The use of different definitions for IFG has led to differences between studies, resulting in noncomparable estimates of prevalence and risk factors for progression to diabetes.

Multiple studies have described differences in the prevalence of IFG using the different criteria. A study estimated the global prevalence of IFG-WHO in people aged 20–79 to be 5.8% [5]. Prevalence also varies by ethnicity; a meta-analysis identified that a greater proportion of Caucasians had IFG compared to Asians (43.9% Caucasian, 29.2% Asian, IFG-WHO; 58.0% Caucasian, 48.1% Asian, IFG-ADA) [6]. These studies suggest that the ADA criterion captures a larger portion of the population. Despite these inconsistencies, studies show that people with IFG by either criterion have an increased risk of progression to diabetes. A meta-analysis including 76,513 participants and a mean follow-up of 11.1 years reported the risk of progression was greater for IFG-WHO (5.54, 95% CI 4.32–7.12) compared to IFG-ADA (4.17, 95% CI 3.36–5.17) [7]. Whilst the risk of progression is greater in IFG-WHO, individuals with IFG-ADA were also at increased risk.

The aim of this study was to determine the prevalence of diabetes and IFG according to ADA and WHO criteria and identify risk factors for the progression to diabetes and regression to normoglycaemia in Australian men.

## 2. Materials and Methods

### 2.1. Population Background

This study used data from male participants of the Geelong Osteoporosis Study (GOS), which randomly recruited residents from the Barwon Statistical Division in south-eastern Australia [8]. An age-stratified cohort of men (ages 20–96 years at baseline) attended three assessment phases. Baseline was conducted between 2001 and 2006 and included 1540 men. The 5-year follow-up was conducted during 2007–2010 and included 978 men, and the 15-year follow-up was finalised in 2022 and included 629 men. Comparable data have already been published for the female cohort [9].

### 2.2. Study Population

Participants had glycaemia status determined at either baseline (*n* = 997) or the 5-year (*n* = 173) follow-up. Other data were drawn from the appropriate assessment phase. For clarity, the term ‘study baseline' will be used to indicate when glycaemia status was first determined.

**Figure 1:**
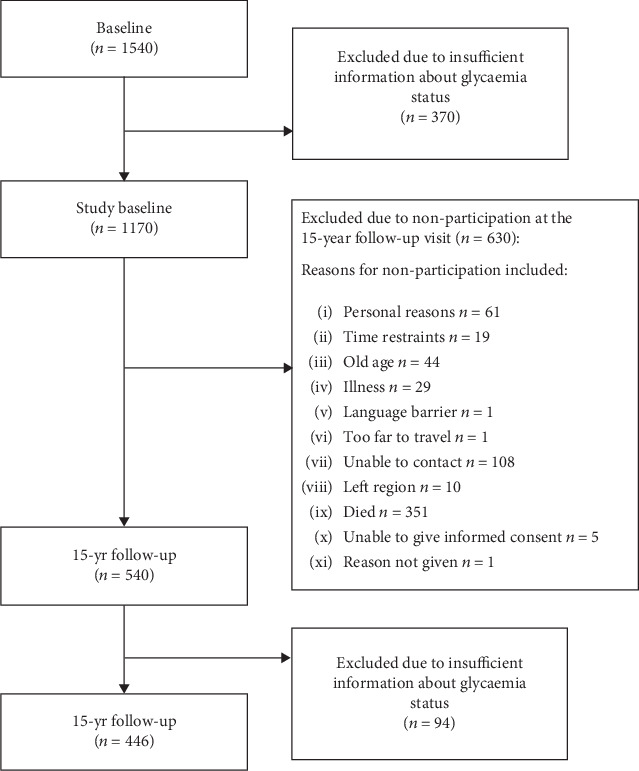
Participant follow-up chart, showing participation in the progression and regression analyses.

Of 1540 men who participated at baseline, glycaemia status was assessed for 1170 (Figure 1). Of these, 446 provided sufficient information to determine glycaemia status at the 15-year follow-up visit; 30 men had diabetes at baseline and thus were excluded from progression to diabetes analysis. Progression to diabetes was defined as a participant classified as having normoglycaemia/IFG at study baseline and then classified as having diabetes at the 15-year follow-up visit; 416 men were included in this analysis. Regression to normoglycaemia was defined as a participant with IFG at study baseline and then classified as having normoglycaemia at the 15-year follow-up visit. The men included in these analyses (92 IFG-ADA and 29 IFG-WHO) did not report use of any antihyperglycaemic medication.

### 2.3. Blood Sampling

After an overnight fast, blood samples were collected at study baseline and stored at −80°C before analyses. Participants were considered to have diabetes if they had FPG ≥ 7.0 mmol/L (hexokinase–glucose-6-phosphate dehydrogenase method), self-reported use of antihyperglycaemic medication and/or self-report of diabetes. Glycated haemoglobin A1c was measured in-house using an ELISA kit for HbA1c (CEA190Hu, CLOUD-CLONE). Further tests were conducted at pathology laboratories and included serum triglycerides (glycerol phosphate oxide method, Abbott Alinity instrument), HDL cholesterol (accelerator selective detergent method, Abbott Alinity instrument), LDL cholesterol (calculated using FriedWald formula), total cholesterol (enzymatic method, Abbott Alinity instrument), glutamic acid decarboxylase antibody, C-peptide (C-peptide chemiluminescence immunoassay) and serum creatinine (enzymatic assay for creatinine). HOMA-IR was calculated as 1.5 + FPG (mmol/L) × fasting C − peptide (pmol/L)/2800, and HOMA-B was calculated as 0.27 × C − peptide (pmol/L)/(FPG (mmol/L) − 3.5) [10].

### 2.4. Clinical Measurements

Height and weight were measured to the nearest ±0.1 cm and ±0.1 kg, respectively. Multiple measures of adiposity included BMI, waist and hip circumference and body fat mass. BMI was calculated as weight (kilogramme)/height (metre)^2^. Waist circumference was measured as the minimum circumference between the lowest rib and the iliac crest to the nearest ±0.5 cm. Hip circumference (centimetre) was measured at the maximum gluteal region. An automated sphygmomanometer (Takeda Medical UA-751) was used to measure seated blood pressure. Body fat mass (kilogramme) and lean mass (kilogramme) were estimated using whole-body dual-energy x-ray absorptiometry (DXA); body fat percent was calculated using fat mass/(fat mass + lean mass) × 100. A Lunar DPX-L (Lunar; Madison, WI, United States) was used for 544 men, and the GE Prodigy (Prodigy; GE Lunar, Madison, WI, United States) was used when the original scanner became outmoded. Fatty liver index was estimated using a prediction formula [11] that included clinical features associated with fatty liver (BMI, waist circumference, triglyceride levels and gamma-glutamyl transferase [activity colorimetric assay]); a higher fatty liver index indicates greater severity.

### 2.5. Questionnaires

Self-reported questionnaires were used to determine physical activity, alcohol consumption, smoking status and use of medications. ‘Very active, active, sedentary, limited, inactive, chair/bedridden and bedfast' were potential responses for physical activity level. These were then categorised as ‘high' for those that were ‘very active' and ‘active' and ‘low' for the remaining groups. The Cancer Council Victoria Food Frequency Questionnaire was used to determine alcohol consumption (grammes per day) [12]. Alcohol consumption was categorised as ‘low' if < 30 g/day and ‘high' if ≥ 30 g/day based upon National Health and Medical Research Council (NHMRC) recommendations [13]. Smoking was defined as current smokers and nonsmokers (included former smokers).

### 2.6. Statistical Analyses

The ADA and WHO classifications of IFG were both used in this study. Normoglycaemia was defined based upon the ADA criterion (FPG < 5.6 mmol/L) [4] for variable statistic values and prevalence analyses.

Histograms were used to investigate the normality of the data. Differences in continuous variables between groups were identified using ANOVA or Kruskal–Wallis tests as appropriate; similarly, Tukey's or Dunn tests were used for post hoc analysis. Differences in discrete variables were determined using the chi-square test (or Fisher's exact test).

Proportions of men with IFG and diabetes were calculated at study baseline. Age-standardised prevalence estimates were calculated using 2006 Australian population data, and 95% confidence intervals were determined. The age ranges for this analysis were 20–29, 30–39, 40–49, 50–59, 60–69, 70–79 and 80+ years.

Multivariable logistic regression modelling was used to determine risk factors for progression to diabetes and regression to normoglycaemia. Those with missing data were excluded from analyses where data were missing. Univariate analyses included each variable individually in a logistic regression model; all variables significantly associated with progression to diabetes (*p* < 0.1) were tested in a multivariable model. To avoid collinearity, measures of body composition were tested in separate models. Similarly, C-peptide, HOMA-IR and HOMA-B were tested in models without FPG due to high correlation. Statin use was associated with age and adiposity; the fatty liver index correlated with HDL cholesterol. There was little correlation found between FPG and HbA1c, and thus, these variables were tested in models together. Adjusting for height in the models containing lean mass did not significantly affect the outcome. Receiver operator characteristic curves were set up for each model; optimal FPG cut points were determined as the point where the difference between sensitivity and specificity was the smallest. All analyses were completed using StataIC 18 (StataCorp 2023. *Stata Statistical Software: Release 18*. College Station, TX: StataCorp LLC).

## 3. Results

### 3.1. Study Baseline Characteristics

Of 1170 participants included in these analyses, 790 (67.5%) had normoglycaemia, 256 (21.9%) had IFG-ADA, 93 (8.0%) had IFG-WHO and 124 (10.6%) had diabetes (three had Type 1 diabetes and positive glutamic acid decarboxylase antibodies). Using FPG ≥ 7.0 mmol/L, 70 men were classified as having diabetes, 89 self-reported diabetes and 76 reported use of antihyperglycaemic medications; many of these criteria overlapped.

Of those using antihyperglycaemic medications, 14 were taking insulin, 47 were taking biguanides, 43 were taking sulfonylureas and 1 was taking a thiazolidinedione.

Across the glycaemia groups, there was a pattern of increasing age, FPG and HOMA-IR; men with diabetes had the highest median age, whereas those in the normoglycaemia group had the lowest (Table 1). This was the opposite for eGFR, decreasing from normoglycaemia to IFG-ADA to diabetes. Men with IFG-ADA or diabetes also had higher mean values for adiposity, systolic blood pressure, serum triglycerides, C-peptide and fatty liver index compared to those with normoglycaemia. The diabetes group was shorter, had higher mean creatinine, lower mean HDL, LDL cholesterol and HOMA-B and had higher statin use compared to normoglycaemia. In contrast, the IFG-ADA group only had lower HDL cholesterol and HOMA-B. No differences were detected in lean mass, smoking status and alcohol consumption. Data relating to those only characterised under the IFG-WHO criterion is available in Table S1.

### 3.2. Prevalence of IFG and Diabetes

**Figure 2:**
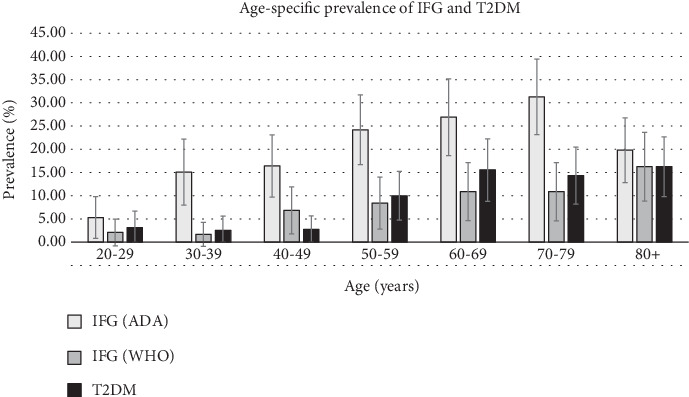
Age-specific prevalence of impaired fasting glucose under the American Diabetes Association definition (light colouring, IFG [ADA]), World Health Organisation definition (dark shading, IFG [WHO]) and diabetes (darkest shading). Error bars represent 95% confidence intervals.

The prevalence of both IFG and diabetes increased with age. Prevalence increased from 5.3% and 2.1% in the 20–29-year age group to 31.3% and 10.9% in the 70–79-year age group for IFG-ADA and IFG-WHO, respectively (Figure 2). For diabetes, prevalence in the 20–29-year age group was 3.2%, increasing to 16.2% in the ≥ 80-year age group.

The age-standardised prevalence for men with IFG-ADA was 18.2% (95% CI 15.7–20.7), IFG-WHO 6.2% (95% CI 4.8–7.6) and 7.3% for diabetes (95% CI 5.8–8.8).

### 3.3. Nonparticipation Statistics

Of 724 men who did not participate in the progression analyses, 630 were lost to follow-up, and 351 deaths occurred over the follow-up, with 129 (40.4%) associated with a cardiovascular event (further reasons provided in Figure 1). The remaining men did not have sufficient data to determine glycaemia status at the 15-year follow-up. These men were older and had higher FPG, systolic blood pressure and diastolic blood pressure, whereas they had lower LDL cholesterol. They also had lower lean mass and weight, were shorter, had higher waist circumference and had a greater prevalence of physical inactivity at study baseline compared to those who did participate (Table S2).

### 3.4. Sample Population Statistics

**Figure 3:**
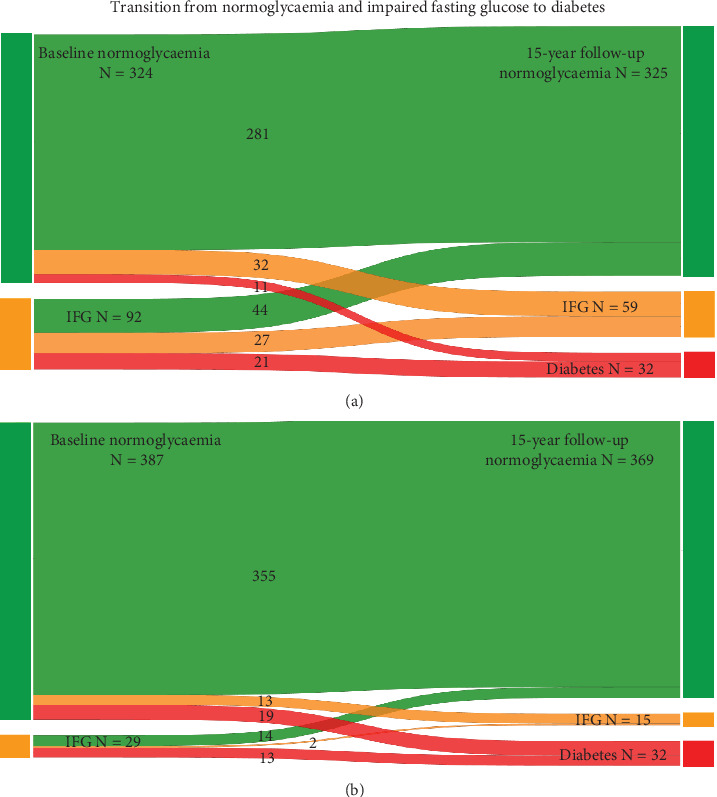
Sankey plot for the transition from normoglycaemia (green) and impaired fasting glucose (IFG) (orange) to diabetes (red) from study baseline to the 15-year follow-up. (a) Transition with IFG using the American Diabetes Association criteria. (b) Transition with IFG using the World Health Organisation criteria. Naqvi (2024). Stata package ‘sankey' version 1.74. Release date 11 June 2024. https://github.com/asjadnaqvi/stata-sankey.

Of 446 participants with sufficient data to determine diabetes status at the 15-year follow-up visit, 324 (72.6%) had normoglycaemia, 92 (20.6%) had IFG-ADA, 29 (6.5%) had IFG-WHO and 30 (6.7%) had diabetes at study baseline (Figure 3). Participants had a median follow-up time of 13.4 (IQR 11.2–13.9) years.

Most men with normoglycaemia at baseline remained in that category (*n* = 281, 86.7%), 32 (9.9%) men progressed to IFG-ADA and 11 (3.4%) men progressed to diabetes. Of 92 men with IFG-ADA at study baseline, approximately half regressed to normoglycaemia (*n* = 44, 47.8%), 27 men (29.3%) remained with IFG-ADA and 21 (22.8%) progressed to diabetes. Of those with IFG-WHO, 13 (44.8%) progressed to diabetes, 14 (48.3%) regressed to normoglycaemia and 2 (6.9%) remained with IFG-WHO. Most participants with diabetes at study baseline remained in this category; one participant was reclassified with IFG. Over a total follow-up time of 5220.9 person-years, 32 men developed diabetes. This corresponds to an age-standardised incidence of 27.8 (95% CI 18.3–37.2) per 1000 person-years.

At study baseline, men who progressed to diabetes were older and shorter and had higher FPG, HbA1c, serum triglycerides, C-peptide, HOMA-IR, fatty liver index, statin use and lower HDL cholesterol. Measures of body composition were also different compared to those who did not progress: greater weight, BMI, waist circumference, body fat mass and body fat percent.

### 3.5. Progression to Type 2 Diabetes Mellitus

Progression to diabetes was assessed in 416 men. Of 32 men who progressed to diabetes, 22 were categorised using FPG ≥ 7.0 mmol/L, 25 self-reported diabetes and 19 reported use of antihyperglycaemic medications; many of these criteria overlapped.

Of those using antihyperglycaemic medications, 15 were taking biguanides, 9 were taking sulfonylureas, 6 were taking DPP-4 inhibitors, 2 were taking SGLT2 inhibitors and 1 was taking a GLP-1 agonist.

FPG, HbA1c, age, serum triglycerides, HDL cholesterol, C-peptide, HOMA-IR, fatty liver index, statin use, weight, height, BMI, waist circumference, body fat mass and body fat percent were different comparing to those who remained without diabetes and those that progressed to diabetes (Table 2). In univariate logistic regression models, these factors were also associated with the progression to diabetes over the follow-up (Table 3). Weight gain over the follow-up was not significantly associated with an increased risk of progression to diabetes.

FPG, HbA1c, age, HDL cholesterol and body fat percent remained significant in multivariable analysis. The likelihood of progression to diabetes was more than six times greater for each 1 mmol/L increase in FPG. For each year of age, there was a 5% increased risk of progressing to diabetes. A higher body fat percent increased the likelihood of progression, whereas having higher HDL cholesterol was associated with a lower risk of progression to diabetes (Table 3).

Men with IFG-ADA had an 8.4 (95% CI 3.9–18.2, *p* < 0.001) times greater likelihood of progressing to diabetes, whereas those with IFG-WHO had a 15.7 (95% CI 6.6–37.4, *p* < 0.001) times greater chance of progressing over the follow-up.

### 3.6. Statin Use in Progression to Diabetes

Whilst significant in bivariate analysis, statin use was not significant with progression to diabetes in multivariable analyses. The correlation with age may have contributed to this. Statin users were older, heavier, had a higher BMI, and a larger body fat mass and percentage. They also had higher fasting glucose, HbA1c, C-peptide, estimated insulin resistance and fatty liver index. LDL cholesterol was lower within this group which may be a result of statin use.

### 3.7. Sensitivity Analysis

The best prediction models included FPG, HbA1c, HDL cholesterol, age and a measure of adiposity (body fat percent) or physical activity when these were not included. Using the receiver operator characteristic curve, a univariate model with FPG produced an area under the curve of 0.784. The optimal FPG cut point was 5.3 mmol/L with a corresponding sensitivity of 68.8%, a specificity of 75.3%, a positive predictive value of 18.8% and a negative predictive value of 96.7%. The model containing age, FPG and HDL cholesterol produced an area under the curve of 0.836 and an optimal FPG cut point of 5.6 mmol/L, corresponding to a sensitivity of 77.4% and a specificity of 77.8%, a positive predictive value of 22.0% and a negative predictive value of 97.7%. This model with HbA1c added produced a cut point of 5.3 mmol/L; similar results were produced by adding body fat percent. When adding both HbA1c and body fat percent, a cut point of 5.2 mmol/L was produced.

### 3.8. Regression to Normoglycaemia

Of 92 men with IFG-ADA at study baseline, those who regressed were younger and had higher HDL cholesterol, lower C-peptide, HOMA-IR, HOMA-B and fatty liver index (Table 4). Systolic blood pressure was lower in those who regressed from the 29 men with IFG-WHO; no factors were associated with regression in this group.

Age and HDL cholesterol were significantly associated with regression in IFG-ADA in multivariable analysis. There was a 5% lower chance of regression with each year of age older. The likelihood of regression increased 1.90-fold for each standard deviation increase in HDL cholesterol (Table 5).

## 4. Discussion

This study identified that the prevalence of IFG is higher than diabetes in Australian men, and physical and clinical characteristics in IFG appear to be intermediate between normoglycaemia and diabetes. FPG, HbA1c, age, HDL cholesterol and body fat percent were significant risk factors for progression to diabetes. A prediction model containing age, FPG, HDL cholesterol and HbA1c predicted an optimal FPG cut point of 5.3 mmol/L for progression to diabetes.

We reported an age-standardised prevalence for IFG-ADA that was higher than diabetes, whereas the prevalence was similar for IFG-WHO. The age-standardised prevalence from the female cohort of the GOS was 31.5% (95% CI 28.4–34.5) for IFG-ADA and 5.6% (95% CI 4.5–6.7) for diabetes [9]. The prevalence of IFG-ADA was lower in men compared to women, whereas the prevalence of diabetes appeared higher in men, albeit the 95% confidence intervals overlapped. An American population-based study reported a prevalence of IFG-ADA of 19.4% (95% CI 16.3–22.4) [14], and similarly, a Canadian study reported a prevalence of 18.1% (95% CI 15.3–21.3) in men [15].

An age-adjusted prevalence of diabetes of 7.9% (95% CI 7.4–8.3) was reported in a US national survey [16], similar to the prevalence in this study at a similar time point. Prevalence of diabetes was estimated to be lower in young Australian men (18–49 years), reporting a prevalence of 3.0% (95% CI 2.5–3.4) [17]. This is expected as the prevalence of diabetes generally increases with age; therefore, they did not capture those diagnosed later in life. Additionally, diabetes status was self-reported, likely underestimating the prevalence, as this condition is often undiagnosed [18].

This study indicated that a higher FPG and HbA1c conferred increased risk of progression to diabetes, whereas lower levels increased probability of regression to normoglycaemia. These findings have been reported in other studies similarly associating higher FPG [9, 19, 20] and higher HbA1c [21, 22] with increased risk of progression to diabetes. Diabetes is a result of an inability to meet insulin demands whether through poor insulin production and/or high insulin resistance [23]. Elevated fasting glucose presents in individuals with sufficient accumulated changes to alter glucose homeostasis, and, as this is progressive, insulin production and resistance become more imbalanced, raising the level of fasting glucose [23].

Insulin resistance is a major component in the pathogenesis of diabetes [24]. In our study, men who progressed to diabetes had higher HOMA-IR. Another study reported insulin resistance to be higher and beta-cell function to be lower in IFG compared to those with normoglycaemia [25]. The study also identified that lower beta-cell function was associated with progression to diabetes after 2 years of follow-up, although this study included participants with IFG and/or impaired glucose tolerance (IGT) in this analysis. HDL cholesterol and dyslipidaemia have been linked to insulin resistance and thus diabetes [26]. This was a major component of progression to diabetes and regression to normoglycaemia in this study. Paprott et al. [27] reported similar results; regression to normoglycaemia was more likely in those with higher HDL cholesterol. Further studies also identified serum triglycerides as a factor in progression to diabetes [19, 28], regression to normoglycaemia [27] or both [9]. Fatty liver index had been associated with progression to diabetes in other studies [29]. These suggest that the greater ability to remove cholesterol from the blood conferred by higher HDL cholesterol may reduce the risk of diabetes and increase the transition back to normal fasting glucose from IFG. Statin use has been associated with a greater rate of progression to diabetes in people with risk factors for diabetes [30]. Whilst statin users had greater mean values for diabetes risk factors, this relationship was not detected in our study.

Age was an important factor for both progression and regression in this study. Similarly, other studies reported that younger ages are associated with regression [31] and older ages with progression [27, 28]. However, progression is not as common above the age of 75 years, which may be due to a healthy survivor effect [32]. Indeed, progression is less common than regression to normoglycaemia and death in older individuals with IFG [22].

Measures of adiposity have consistently been associated with progression to diabetes. In this study, body fat percentage was identified as a risk factor. BMI appears to be the most common measure associated with progression, with multiple studies reporting this association [27, 28, 31, 32]; greater waist circumference has also been associated with progression [27, 31]. Adiposity may be linked with insulin resistance, a key component in the pathogenesis of diabetes, promoting progression to diabetes. Lower lean mass was reported to be associated with regression in women [9]. However, women who regressed to normoglycaemia had a higher lean mass relative to body weight; higher body weight may increase absolute lean mass, creating this association, hinting at a relationship between body composition and regression similar to the association with body fat percent and progression reported in this study.

This study identified a FPG of between 5.2 and 5.6 mmol/L to be optimal cut points for predicting the progression to diabetes in models containing risk factors for diabetes. This is consistent with the ADA criterion for IFG and provides evidence for the use over the WHO criterion. As expected in this study, more people are identified using the ADA criterion and thus identified more that progressed to diabetes over the follow-up. This is similar to other studies [14, 15, 33]. Using receiver operator characteristic curve analysis, Lee et al. [7] reported a high specificity and low sensitivity (93.8 and 39.1) for IFG-WHO compared to more balanced specificity and sensitivity (77.0 and 64.1) for IFG-ADA, similar to this study. They also reported that an optimal FPG cut point for attributing diabetes risk after 5 years was 5.6 mmol/L. This creates a balancing act between identifying the most individuals at risk of developing diabetes and not categorising those at low risk as having high risk. The classification of progressing to diabetes in the prediction models is based upon a threshold of risk from factors in the model. Glucose screening for those with multiple risk factors for diabetes or associated with progression to diabetes seems logical to identify those at greatest risk of developing diabetes. Whilst there is evidence that a combination of exercise and dietary intervention can be effective at preventing progression to diabetes, further investigation is required [34].

Those with IFG-WHO were more likely to progress to diabetes compared to IFG-ADA criterion (OR 15.7, 6.6–37.4 vs. 8.4, 3.9–18.2) in this study, although both had a greater chance of progression compared to normoglycaemia. Similar risks of progression to diabetes in both criteria (15.4 WHO and 7.0 ADA) were reported by Schmidt et al. [35]. They noted that IFG-ADA identified more people who progressed, but a smaller portion of this group progressed compared to IFG-WHO. Furthermore, the risk of progression in those categorised with only IFG-ADA was 4.7 (95% CI 3.8–6.0) times greater compared to those with normoglycaemia [33]. A meta-analysis reported greater risk of progression to diabetes in both IFG criteria [21]. This indicates that IFG-ADA is important as they still carry an increased risk of progression and thus the potential complications associated with diabetes.

This study had both strengths and limitations. The strengths are that the analyses included a large sample of men across a wide age range, who were randomly selected from the general population and had not been selected on the basis of disease. The criteria used to categorise men into glycaemia groups were robust, including FPG, self-report of the condition or antihyperglycaemic medications. This provides redundancy as people using antihyperglycaemic medications may not be classified using FPG alone; this is important as a single blood test was conducted at each follow-up rather than multiple tests to limit participant burden. Body composition measures were objective, with whole-body DXA scans used to determine fat and lean mass values; weight and height were also measured. Biochemical data was also available for the analysis.

Limitations of this study include that the analyses included men only (primarily Caucasian, 98%); therefore, the results may not be generalisable to other groups, including women. Comparable data in women from the GOS have already been published. The lack of IGT mean results are not comparable with other studies that utilised IGT or a combination of IFG and IGT to indicate prediabetes. Some data were collected through self-report, and this may have been affected by recall bias. There may have been differential loss to follow-up for the analysis which identified risk factors for progression to diabetes; people likely to develop diabetes may have died over the follow-up, and this may have led to an underestimate of the conversion. Indeed, these differences were presented in the results section. Furthermore, there were differences in the sample sizes between the normoglycaemia (*n* = 790), IFG (*n* = 256) and diabetes (*n* = 124) groups and the groups of those that progressed to diabetes (*n* = 32) and those that remained without diabetes (*n* = 384); thus, this may be at risk of bias. Bias of this nature is concerning when variances are unequal, which is not the case. However, statistical power may be restricted by the size of the smallest groups.

## 5. Conclusions

This study may help validate the use of the ADA classification of IFG as a prediction model containing age, FPG, HDL cholesterol and HbA1c, which predicted an optimal FPG cut point of 5.3 mmol/L for predicting the progression to diabetes in men from the GOS. Whilst those classified under the ADA criterion have a lower chance of progressing to diabetes, they still carry an excess risk of progression. Risk factors identified are potentially modifiable and thus could be a target for prevention strategies for diabetes.

## Figures and Tables

**Table 1:** Descriptive characteristics for participants with normoglycaemia, impaired fasting glucose (IFG) or diabetes at study baseline (2001–2006). Data presented as mean ± SD, median (IQR) or *n* (%).

	**Normoglycaemia (** **n** = 790**)**	**IFG-ADA (** **n** = 256**)**	**Diabetes (** **n** = 124**)**	**p** ** value**
Age (year)	57.0 (41.2–74.6)	67.7 (53.0–76.8)	70.3 (61.1–75.5)	< 0.001
Weight (kg)	81.2 ± 13.9	86.9 ± 15.3	85.7 ± 14.3	< 0.001
Height (cm)	174.7 ± 7.4	174.1 ± 7.1	172.5 ± 6.9	0.006
BMI (kg/m^2^)	26.6 ± 4.0	28.6 ± 4.3	28.8 ± 4.6	< 0.001
Waist circumference (cm)	96.1 ± 10.4	102.3 ± 11.7	103.6 ± 12.0	< 0.001
Hip circumference (cm)	99.8 ± 8.4	103.6 ± 9.0	104.7 ± 9.5	< 0.001
Systolic blood pressure (mmHg)	135.4 ± 17.0	141.3 ± 17.8	142.9 ± 20.0	< 0.001
Diastolic blood pressure (mmHg)	84.9 ± 11.2	87.7 ± 14.6	85.4 ± 14.6	0.011
Fat mass (kg)	20.8 ± 8.0	24.6 ± 8.4	24.4 ± 8.4	< 0.001
Lean mass (kg)	57.4 ± 7.4	58.6 ± 7.4	57.6 ± 7.2	0.102
Body fat percentage	25.9 ± 7.2	28.9 ± 6.5	29.1 ± 9.2	< 0.001
Smoking	108 (13.7)	27 (10.6)	13 (10.5)	0.371
High alcohol consumption	169 (21.8)	66 (26.7)	19 (16.2)	0.068
Physical inactivity	180 (22.8)	84 (32.8)	45 (36.3)	< 0.001
FPG (mmol/L)	4.91 ± 0.47	5.98 ± 0.34	7.68 ± 2.12	< 0.001
HbA1c (*μ*g/mL)	56.7 (46.1–117.2)	56.5 (46.7–78.8)	65.0 (47.5–121.2)	0.357
C-peptide (nmol/L)	0.56 (0.42–0.75)	0.78 (0.59–1.01)	0.75 (0.51–1.00)	< 0.001
HOMA-IR	1.86 ± 0.17	2.09 ± 0.27	2.23 ± 0.48	< 0.001
HOMA-B	42.7 ± 43.0	30.1 ± 12.7	23.3 ± 25.5	< 0.001
Serum triglycerides (mmol/L)	1.47 ± 0.81	1.86 ± 1.04	1.77 ± 0.79	< 0.001
HDL cholesterol (mmol/L)	1.33 ± 0.29	1.26 ± 0.28	1.22 ± 0.28	< 0.001
LDL cholesterol (mmol/L)	3.14 ± 0.83	3.13 ± 0.87	2.78 ± 0.28	< 0.001
Creatinine	85.3 ± 20.7	87.1 ± 19.5	92.0 ± 20.1	0.005
Fatty liver index	48.7 ± 26.3	66.5 ± 24.4	67.1 ± 25.1	< 0.001
Statin use	106 (13.4)	50 (19.5)	46 (37.1)	< 0.001

*Note:* Missing data: weight, height, BMI *n* = 3, waist/hip circumference *n* = 26, systolic/diastolic blood pressure *n* = 70, fat/lean mass *n* = 16, alcohol consumption *n* = 30, HbA1c *n* = 21, serum triglycerides, HDL cholesterol *n* = 22 and LDL cholesterol *n* = 36.

Abbreviations: BMI, body mass index; FPG, fasting plasma glucose; HbA1c, glycated haemoglobin A1c; HDL, high-density lipoprotein; HOMA-B, homeostatic model assessment for beta-cell dysfunction; HOMA-IR, homeostatic model assessment for insulin resistance; IFG, impaired fasting glucose; LDL, low-density lipoprotein.

**Table 2:** Descriptive statistics for men included in the progression analysis. Data shown as mean ± SD, median (IQR) or *n* (%).

**Factors**	**Remained without diabetes (** **n** = 384**)**	**Progressed to diabetes (** **n** = 32**)**	**p** ** value**
Age (year)	51.3 ± 13.5	58.9 ± 10.8	0.002
Weight (kg)	83.6 ± 13.1	89.7 ± 14.5	0.012
Height (cm)	176.2 ± 6.8	173.0 ± 6.4	0.009
BMI (kg/m^2^)	26.9 ± 3.8	29.9 ± 3.9	< 0.001
Waist circumference (cm)	96.4 ± 10.6	103.8 ± 10.8	< 0.001
Hip circumference (cm)	100.6 ± 8.9	103.8 ± 8.0	0.052
Systolic blood pressure (mmHg)	131.8 ± 17.5	137.7 ± 18.5	0.073
Diastolic blood pressure (mmHg)	80.8 ± 11.0	82.6 ± 11.0	0.365
Fat mass (kg)	21.1 ± 8.0	27.0 ± 7.9	< 0.001
Lean mass (kg)	59.3 ± 6.9	58.8 ± 6.7	0.621
Body fat percentage	25.6 ± 7.0	30.9 ± 5.6	< 0.001
Smoking	47 (12.2)	3 (9.3)	0.632
High alcohol consumption	91 (24.0)	9 (29.0)	0.531
Physical inactivity	64 (16.7)	9 (28.13)	0.102
Fasting plasma glucose (mmol/L)	5.10 ± 0.53	5.72 ± 0.91	< 0.001
HbA1c (*μ*g/mL)	39.1 (51.3–76.6)	59.9 (43.0–126.6)	0.114
C-peptide (nmol/L)	0.59 ± 0.25	0.95 ± 0.41	< 0.001
HOMA-IR	1.86 ± 0.17	2.15 ± 0.35	< 0.001
HOMA-B	36.4 ± 22.5	36.6 ± 14.2	0.968
Serum triglycerides (mmol/L)	1.54 ± 0.88	1.95 ± 1.14	0.014
HDL cholesterol (mmol/L)	1.33 ± 0.28	1.16 ± 0.18	0.002
LDL cholesterol (mmol/L)	3.29 ± 0.80	3.34 ± 0.90	0.730
Creatinine	81.5 ± 12.0	84.6 ± 13.0	0.161
Fatty liver index	50.6 ± 26.3	73.0 ± 21.7	< 0.001
Statin use	31 (8.1)	9 (28.1)	< 0.001

*Note:* Missing data: waist/hip circumference *n* = 9, systolic/diastolic blood pressure *n* = 10, fat/lean mass *n* = 5, alcohol consumption *n* = 6, HbA1c *n* = 1 and serum triglycerides *n* = 2.

Abbreviations: BMI, body mass index; FPG, fasting plasma glucose; HbA1c, glycated haemoglobin A1c; HDL, high-density lipoprotein; HOMA-B, homeostatic model assessment for beta-cell dysfunction; HOMA-IR, homeostatic model assessment for insulin resistance; IFG, impaired fasting glucose; LDL, low-density lipoprotein.

**Table 3:** Univariable and multivariable logistic regression analysis with odds ratios for the progression to Type 2 diabetes mellitus. All characteristics were measured with fasting plasma glucose. Data presented as OR (95% CI).

**Factor**	**Univariable analysis**	**Model 1**	**Model 2**	**Model 3**	**Model 4**
Fasting plasma glucose (mmol/L)	6.31 (3.23–12.34)⁣^∗∗∗^	6.44 (2.97–13.94)⁣^∗∗∗^	5.34 (2.45–11.67)⁣^∗∗∗^	—	—
HbA1c^a^	1.34 (1.00–1.79)⁣^∗^	1.52 (1.08–2.13)⁣^∗^	1.67 (1.17–2.37)⁣^∗∗^	1.33 (0.96–1.83)	1.36 (0.98–1.90)
C-peptide (nmol/L)^b^	2.50 (1.81–3.45)⁣^∗∗∗^	—	—	2.11 (1.49–2.99)⁣^∗∗∗^	—
HOMA-IR^c^	2.56 (1.86–3.51)⁣^∗∗∗^	—	—	—	2.22 (1.57–3.14)⁣^∗∗∗^
HOMA-B	1.00 (0.98–1.02)	—	—	—	—
Age (year)	1.04 (1.02–1.08)⁣^∗∗^	1.05 (1.01–1.09)⁣^∗∗^	1.04 (1.00–1.09)⁣^∗^	1.05 (1.01–1.09)⁣^∗^	1.05 (1.01–1.09)⁣^∗^
Systolic blood pressure (mmHg)	1.01 (1.00–1.04)	1.01 (0.98–1.03)	—	—	—
Diastolic blood pressure (mmHg)	1.01 (0.98–1.04)	—	—	—	—
Smoking	0.76 (0.22–2.61)	—	—	—	—
High alcohol consumption	1.31 (0.57–2.96)	—	—	—	—
Physical inactivity	1.69 (0.72–3.95)	—	—	—	—
Serum triglycerides	1.43 (1.06–1.93)⁣^∗^	0.85 (0.55–1.32)	—	—	—
Standardised HDL^c^	0.47 (0.29–0.75)⁣^∗∗^	0.49 (0.30–0.80)⁣^∗∗^	0.49 (0.29–0.81)⁣^∗∗^	0.61 (0.37–1.02)	0.62 (0.37–1.02)
LDL cholesterol (mmol/L)	1.09 (0.68–1.72)	—	—	—	—
Creatinine	1.02 (0.99–1.05)	—	—	—	—
Fatty liver index	1.04 (1.02–1.06)⁣^∗∗∗^	1.02 (1.00–1.04)	—	—	—
Statin use	4.46 (1.90–10.46)⁣^∗∗∗^	2.20 (0.83–5.84)	—	—	—
Measures of body composition^d^					—
Weight (kg)	1.03 (1.01–1.06)⁣^∗^	—	1.01 (0.98–1.04)	—	—
Height (cm)	0.93 (0.88–0.98)⁣^∗∗^	—	0.94 (0.88–0.1.00)	—	—
BMI (kg/m^2^)	1.17 (1.08–1.27)⁣^∗∗∗^	—	1.08 (0.98–1.19)	—	—
Waist circumference (cm)	1.06 (1.02–1.09)⁣^∗∗∗^	—	1.02 (0.98–1.06)	—	—
Hip circumference (cm)	1.04 (1.00–1.08)	—	—	—	—
Body fat mass (kg)	1.09 (1.04–1.13)⁣^∗∗∗^	—	1.05 (1.00–1.11)	—	—
Body lean mass (kg)	0.99 (0.94–1.04)	—	—	—	—
Body fat percent	1.13 (1.07–1.21)⁣^∗∗∗^	—	1.10 (1.01–1.18)⁣^∗^	—	—

*Note:* Model 1: FPG, HbA1c, age and HDL cholesterol as the main significant variables (other variables shown were tested but were not significant). Model 2: Model 1 plus body fat percent (other variables shown were tested but were not significant). Model 3: HbA1c, age, HDL cholesterol and C-peptide. Model 4: HbA1c, age, HDL cholesterol and HOMA-IR.

Abbreviations: BMI, body mass index; HbA1c, glycated haemoglobin A1c; HDL, high-density lipoprotein; HOMA-B, homeostatic model assessment for beta-cell dysfunction; HOMA-IR, homeostatic model assessment for insulin resistance; IFG, impaired fasting glucose; LDL, low-density lipoprotein.

^a^HbA1c was log transformed because it was not normally distributed.

^b^C-peptide and HOMA-IR were all highly correlated with fasting plasma glucose; multivariable models for these variables included age, HDL cholesterol and HbA1c.

^c^Standardised to z-scores by subtracting the mean then dividing by standard deviation, replaced HDL cholesterol, HOMA-IR and C-peptide within the model.

^d^Measures of body composition were correlated and thus tested in the multivariable model one at a time. The ORs describe the association between each measure and the outcome (progression to diabetes) when included in a model with the other nonbody composition variables (fasting glucose level, age, blood pressure, smoking status and high alcohol consumption).

⁣^∗^Significance at the 0.05 level, ⁣^∗∗^significance at the 0.01 level and ⁣^∗∗∗^significance at < 0.001.

**Table 4:** Descriptive statistics for men with IFG-ADA included in the regression analysis. Data shown as mean ± SD, median (IQR) or *n* (%).

	**Remained with IFG (** **n** = 48**)**	**Regressed to normoglycaemia (** **n** = 44**)**	**p** ** value**
Age (year)	58.3 ± 9.7	52.8 ± 12.1	0.018
Weight (kg)	90.7 ± 12.3	87.0 ± 17.6	0.243
Height (cm)	175.4 ± 6.1	175.0 ± 6.7	0.764
BMI (kg/m^2^)	29.5 ± 4.0	28.3 ± 4.7	0.199
Waist circumference (cm)	103.7 ± 10.8	100.7 ± 12.9	0.227
Hip circumference (cm)	104.9 ± 8.6	102.6 ± 10.0	0.245
Systolic blood pressure (mmHg)	138.3 ± 18.7	137.5 ± 20.9	0.842
Diastolic blood pressure (mmHg)	84.5 ± 11.0	85.8 ± 13.8	0.614
Fat mass (kg)	26.6 ± 8.5	23.6 ± 8.1	0.097
Lean mass (kg)	60.4 ± 6.0	58.8 ± 7.5	0.248
Body fat percent	30.0 ± 6.5	28.1 ± 5.7	0.148
Smoking	5 (10.4)	8 (18.2)	0.285
High alcohol consumption	13 (28.9)	19 (44.2)	0.136
Physical inactivity	10 (20.8)	8 (18.2)	0.749
Fasting glucose (mmol/L)	6.00 ± 0.37	5.88 ± 0.31	0.086
HbA1c (*μ*g/mL)	52.6 (42.0–72.9)	49.6 (39.0–56.3)	0.118
C-peptide (nmol/L)	0.90 ± 0.40	0.70 ± 0.29	0.006
HOMA-IR	2.15 ± 0.31	1.99 ± 0.22	0.007
HOMA-B	31.9 ± 12.5	26.0 ± 9.9	0.014
Serum triglycerides (mmol/L)	2.11 ± 1.25	1.88 ± 0.96	0.324
HDL cholesterol (mmol/L)	1.20 ± 0.24	1.33 ± 0.23	0.013
LDL cholesterol (mmol/L)	3.27 ± 0.81	3.46 ± 0.75	0.254
Creatinine	84.1 ± 12.4	79.1 ± 9.1	0.033
Fatty liver index	73.7 ± 20.7	63.3 ± 24.5	0.033
Statin use	10 (20.8)	3 (6.8)	0.054

*Note:* Missing data: waist/hip circumference *n* = 2, systolic/diastolic blood pressure *n* = 3, fat/lean mass *n* = 1, alcohol consumption *n* = 4 and LDL cholesterol *n* = 5.

Abbreviations: BMI, body mass index; FPG, fasting plasma glucose; HbA1c, glycated haemoglobin A1c; HDL, high-density lipoprotein; HOMA-B, homeostatic model assessment for beta-cell dysfunction; HOMA-IR, homeostatic model assessment for insulin resistance; IFG, impaired fasting glucose; LDL, low-density lipoprotein.

**Table 5:** Univariable and multivariable logistic regression analysis with odds ratios for the regression from IFG-ADA to normoglycaemia. All characteristics were concurrent with measures of fasting plasma glucose.

**Factor**	**Univariate analysis OR (95% CI)**	**p** ** value**	**Multivariable analysis OR (95% CI)**	**p** ** value**
Fasting glucose level	0.33 (0.09–1.18)	0.089	0.48 (0.12–1.95)	0.304
HbA1c^a^	0.84 (0.51–1.38)	0.483	—	—
C-peptide (nmol/L)^b^	0.51 (0.31–0.86)	0.011	0.66 (0.39–1.13)	0.127
HOMA-IR^b^	0.51 (0.30–0.86)	0.011	0.65 (0.38–1.13)	0.128
HOMA-B	0.95 (0.91–0.99)	0.018	0.97 (0.93–1.01)	0.174
Age (year)	0.95 (0.92–0.99)	0.022	0.26 (0.04–1.54)	0.137
Systolic blood pressure (mmHg)	0.99 (0.98–1.02)	0.840	—	—
Diastolic blood pressure (mmHg)	1.01 (0.98–1.04)	0.611	—	—
Smoking	1.91 (0.57–6.36)	0.291	—	—
High alcohol consumption	1.95 (0.81–4.71)	0.138	—	—
Physical inactivity	0.84 (0.30–2.38)	0.749	—	—
Serum triglycerides	0.82 (0.56–1.21)	0.325	—	—
Standardised HDL^b^	1.73 (1.11–2.71)	0.016	1.90 (1.16–3.09)	0.010
LDL cholesterol (mmol/L)	1.38 (0.80–2.39)	0.252	—	—
Creatinine	0.96 (0.92–1.00)	0.038	0.95 (0.91–1.00)	0.044
Fatty liver index	0.98 (0.96–1.00)	0.036	0.98 (0.96–1.01)	0.149
Statin use	0.28 (0.07–1.09)	0.066	—	—
Measures of body composition^c^				
Weight (kg)	0.98 (0.96–1.01)	0.243	—	—
Height (cm)	0.99 (0.92–1.06)	0.761	—	—
BMI (kg/m^2^)	0.94 (0.85–1.03)	0.201	—	—
Waist circumference (cm)	0.98 (0.94–1.01)	0.228	—	—
Hip circumference (cm)	0.97 (0.93–1.02)	0.247	—	—
Body fat mass (kg)	0.96 (0.91–1.01)	0.101	—	—
Body lean mass (kg)	0.96 (0.90–1.03)	0.246	—	—
Body fat percent	0.95 (0.89–1.02)	0.153	—	—

Abbreviations: BMI, body mass index; HbA1c, glycated haemoglobin A1c; HDL, high-density lipoprotein; HOMA-B, homeostatic model assessment for beta-cell dysfunction; HOMA-IR, homeostatic model assessment for insulin resistance; IFG, impaired fasting glucose; LDL, low-density lipoprotein.

^a^HbA1c was log transformed because it was not normally distributed.

^b^Standardised to z-scores by subtracting the mean then dividing by standard deviation.

^c^Measures of body composition were correlated and thus tested in the multivariable model one at a time. The ORs describe the association between each measure and the outcome (progression to diabetes) when included in a model with the other nonbody composition variables (fasting glucose level, age, blood pressure, smoking status and high alcohol consumption).

## Data Availability

Some or all datasets generated during and/or analysed during the current study are not publicly available but are available from the corresponding author on reasonable request.

## Supporting Information

Supporting Information 1 Table S1: Descriptive characteristics for participants with impaired fasting glucose under the WHO criteria at study baseline.

Supporting Information 2 Table S2: Descriptive characteristics of individuals lost to follow-up.
