# Supplementary material for: Risk Factors for the Progression or Regression to Diabetes or Normoglycaemia for Men with Impaired Fasting Glucose
Source: J Diabetes Res. 2025 Oct 10;2025:9926306. doi: 10.1155/jdr/9926306 (PMC12534155; doi:10.1155/jdr/9926306)
Supplement: Supporting Information 1 — Table S1: Descriptive characteristics for participants with impaired fasting glucose under the WHO criteria at study baseline. [file 9926306.f1.docx]

Supplementary table 1: Descriptive characteristics for participants with impaired fasting glucose under the WHO criteria at study baseline. Data presented as mean±SD, median (IQR) or n (%).

| **Factor** | IFG-WHO (n=93) |
| --- | --- |
| Age (y) | 68.3 (55.1-78.1) |
| Weight (kg) | 89.9 ± 15.3 |
| Height (cm) | 174.9 ± 8.1 |
| BMI (kg/m^2^) | 29.3 ± 4.2 |
| Waist circumference (cm) | 105.1 ± 11.3 |
| Hip circumference (cm) | 105.2 ± 8.9 |
| Systolic blood pressure (mmHg) | 144.0 ± 16.7 |
| Diastolic blood pressure (mmHg) | 88.8 ± 14.2 |
| Fat mass (kg) | 26.7 ± 7.5 |
| Lean mass (kg) | 59.2 ± 7.5 |
| Body fat percentage | 30.7 ± 5.2 |
| Smoking | 6 (6.45) |
| High alcohol consumption | 24 (26.4) |
| Physical inactivity | 34 (36.5) |
| ­­FPG (mmol/L) | 6.37 ± 0.23 |
| HbA1c (µg/mL) | 57.1 (46.3-78.6) |
| C-peptide (nmol/L) | 0.87 (0.69-1.08) |
| HOMA-IR | 2.21 ± 0.28 |
| HOMA-B | 29.3 ± 11.0 |
| Serum Triglycerides (mmol/L) | 1.98 ± 1.14 |
| HDL-cholesterol (mmol/L) | 1.22 ± 0.23 |
| LDL-cholesterol (mmol/L) | 3.12 ± 0.90 |
| Creatinine | 88.2 ± 15.8 |
| Fatty liver index | 71.7 ± 22.2 |
| Statin use | 19 (20.4) |

Abbreviations: IFG=Impaired fasting glucose, BMI=Body mass index, FPG=Fasting plasma glucose, HbA1c=Glycated Haemoglobin A1c, HOMA-IR=Homeostatic model assessment for insulin resistance, HOMA-B=Homeostatic model assessment for beta-cell dysfunction, HDL=High density lipoprotein, LDL=Low density lipoprotein.

Missing data: waist/Hip circumference n=3, systolic/diastolic blood pressure n=8, fat/lean mass/body fat percent n=1, high alcohol consumption n=2, LDL-cholesterol n=5, fatty liver n=3.
