# Supplementary material for: Risk Factors for the Progression or Regression to Diabetes or Normoglycaemia for Men with Impaired Fasting Glucose
Source: J Diabetes Res. 2025 Oct 10;2025:9926306. doi: 10.1155/jdr/9926306 (PMC12534155; doi:10.1155/jdr/9926306)
Supplement: Supporting Information 2 — Table S2: Descriptive characteristics of individuals lost to follow-up. [file 9926306.f2.docx]

Supplementary table 2: Descriptive characteristics of individuals lost to follow-up. Data presented as mean±SD, median (IQR) or n (%).

| **Factors** | Non-participants (n=724) |
| --- | --- |
| Age (y) | 71.5 (50.6-80.8) |
| Weight (kg) | 82.1 ± 15.2 |
| Height (cm) | 173.4 ± 7.4 |
| BMI (kg/m^2^) | 27.3 ± 4.4 |
| Waist circumference (cm) | 98.8 ±11.6 |
| Hip circumference (cm) | 101.2 ± 8.9 |
| Systolic blood pressure (mmHg) | 139.0 ± 18.3 |
| Diastolic blood pressure (mmHg) | 85.0 ± 13.1 |
| Fat mass (kg) | 22.2 ± 8.4 |
| Lean mass (kg) | 56.6 ±7.5 |
| Body fat percentage | 27.4 ± 7.2 |
| Smoking | 96 (13.3) |
| High alcohol consumption | 152 (21.7) |
| Physical inactivity | 231 (31.9) |
| ­­FPG (mmol/L) | 5.47 ± 1.23 |
| HbA1c (µg/mL) | 63.8 (48.9-118.8) |
| C-peptide (nmol/L) | 0.71 ± 0.35 |
| HOMA-IR | 1.97 ± 0.29 |
| HOMA-B | 39.5 ± 44.2 |
| Serum Triglycerides (mmol/L) | 1.58 ± 0.85 |
| HDL-cholesterol (mmol/L) | 1.30 ± 0.29 |
| LDL-cholesterol (mmol/L) | 3.00 ± 0.85 |
| Creatinine | 83.5 ± 23.8 |
| Fatty liver index | 55.1 ± 27.2 |
| Statin use | 151 (20.9) |

Abbreviations: IFG=Impaired fasting glucose, BMI=Body mass index, FPG=Fasting plasma glucose, HbA1c=Glycated Haemoglobin A1c, HOMA-IR=Homeostatic model assessment for insulin resistance, HOMA-B=Homeostatic model assessment for beta-cell dysfunction, HDL=High density lipoprotein, LDL=Low density lipoprotein.

Missing data: weight/height/BMI n=3, waist/Hip circumference n=17, systolic/diastolic blood pressure n=80, fat/lean mass/body fat percent n=9, high alcohol consumption n=22, FPG n=17, HbA1c n=19, C-peptide n=24, HOMA-IR/HOMA-B n=25, serum triglycerides n=20, HDL-cholesterol n=20, LDL-cholesterol n=25, creatinine n=18, fatty liver n=30.
